# Multiple synchronous sites of origin of vestibular schwannomas in neurofibromatosis Type 2

**DOI:** 10.1136/jmedgenet-2015-103050

**Published:** 2015-06-23

**Authors:** Stavros M Stivaros, Anat O Stemmer-Rachamimov, Robert Alston, Scott R Plotkin, Joseph B Nadol, Alicia Quesnel, Jennifer O'Malley, Gillian A Whitfield, Martin G McCabe, Simon R Freeman, Simon K Lloyd, Neville B Wright, John-Paul Kilday, Ian D Kamaly-Asl, Samantha J Mills, Scott A Rutherford, Andrew T King, D Gareth Evans

**Affiliations:** 1 Academic Unit of Paediatric Radiology, Royal Manchester Children's Hospital, Central Manchester University Hospitals NHS Foundation Trust, Manchester Academic Health Science Centre, Manchester, UK; 2 Centre for Imaging Sciences, Institute of Population Health, University of Manchester, Manchester, UK; 3 Children's Brain Tumour Research Network, Royal Manchester Children's Hospital, Manchester, UK; 4 Department of Pathology, Massachusetts General Hospital and Harvard Medical School, Boston, Massachusetts, USA; 5 National Drug Evidence Centre (NDEC), Centre for Epidemiology, Institute of Population Health, University of Manchester, Manchester, UK; 6 Department of Neurology, Massachusetts General Hospital and Harvard Medical School, Boston, Massachusetts, USA; 7 Department of Otolaryngology, Massachusetts Eye and Ear Infirmary, Boston, Massachusetts, USA; 8 Radiotherapy Related Research, The Christie NHS Foundation Trust, Manchester, UK; 9 Centre for Paediatric, Teenage and Young Adult Cancer, Institute of Cancer Sciences, University of Manchester, Manchester, UK; 10 Department of Otolaryngology, Manchester Royal Infirmary, Central Manchester University Hospitals NHS Foundation Trust, Manchester Academic Health Science Centre, Manchester, UK; 11 Department of Paediatric Neurosurgery, Royal Manchester Children's Hospital, Central Manchester University Hospitals NHS Foundation Trust, Manchester Academic Health Science Centre, Manchester, UK; 12 Department of Neuroradiology, Salford NHS Foundation Trust Hospital, Salford, Greater Manchester, UK; 13 Department of Neurosurgery, Salford Royal Hospital, Salford, Greater Manchester, UK; 14 Department of Genomic Medicine, St. Mary's Hospital, Central Manchester University Hospitals NHS Foundation Trust, Manchester Academic Health Science Centre, Manchester, UK

**Keywords:** Cancer: CNS, Cancer: head and neck, Clinical genetics, Diagnostics, Neurology

## Abstract

**Background:**

Neurofibromatosis Type 2 (NF2) is a dominantly inherited tumour syndrome with a phenotype which includes bilateral vestibular (eighth cranial nerve) schwannomas. Conventional thinking suggests that these tumours originate at a single point along the superior division of the eighth nerve.

**Methods:**

High resolution MRI was performed in children genetically proven to have NF2. The superior vestibular nerve (SVN) and inferior vestibular nerve (IVN) were visualised along their course with points of tumour origin calculated as a percentage relative to the length of the nerve.

**Results:**

Out of 41 patients assessed, 7 patients had no identifiable eighth cranial nerve disease. In 16 patients there was complete filling of the internal auditory meatus by a tumour mass such that its specific neural origin could not be determined. In the remaining 18 cases, 86 discrete separate foci of tumour origin on the SVN or IVN could be identified including 23 tumours on the right SVN, 26 tumours on the right IVN, 18 tumours on the left SVN and 19 tumours on the left IVN.

**Discussion:**

This study, examining the origins of vestibular schwannomas in NF2, refutes their origin as being from a single site on the transition zone of the superior division of the vestibular nerve. We hypothesise a relationship between the number of tumour foci, tumour biology and aggressiveness of disease. The development of targeted drug therapies in addition to bevacizumab are therefore essential to improve prognosis and quality of life in patients with NF2 given the shortcomings of surgery and radiation treatments when dealing with the multifocality of the disease.

## Background

Neurofibromatosis Type 2 (NF2) is a dominantly inherited tumour syndrome affecting approximately 1 in 33 000 live births.^1^ It has a neuroradiological phenotype characterised by the development of tumours of the central and peripheral nervous system including schwannomas, meningiomas and ependymomas.^2^ ^3^ While schwannomas can be present on any of the cranial nerves (except the optic), the classic finding in NF2 is of bilateral vestibular (eighth cranial nerve) schwannomas (VS). As such, the radiological finding of bilateral VS is the main diagnostic criterion for NF2,^3^ ^4^ although the reason for the predilection for the vestibular nerve remains unexplained.

**Figure 1:**
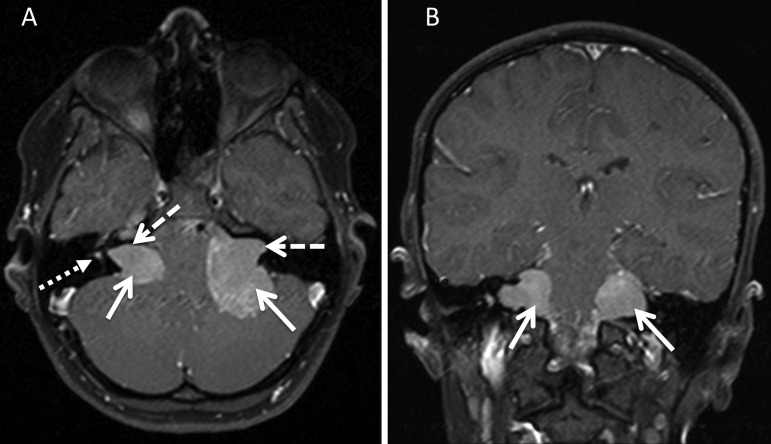
T1 spin echo sequences (axial—A and coronal—B), both with fat saturation and contrast administration showing the typical imaging phenotype of a patient with neurofibromatosis Type 2 . There are bilateral vestibular schwannomas that have a component within the internal auditory meatus (dashed arrow) as well as within the cerebellopontine angle (CPA, solid arrows). These components are in continuity and thought to represent one tumour mass. Further discrete disease is seen in the right vestibule (dotted arrow). The coronal sequence again shows the bilateral CPA angle components of the tumours compressing the brainstem (white arrows).

The imaging of VS has been the subject of progressive scrutiny especially given the recent development of anti-angiogenic treatments such as the antivascular endothelial growth factor A antibody bevacizumab.^5^ Overall tumour size can be an important factor with regards to tumour assessment while on therapy and when determining a patient's eligibility for treatment. Recent work has demonstrated that simple linear measurement of these tumours is not a parameter for satisfactory assessment given their morphological appearance extending out of the internal auditory meatus (IAM) with an associated large cerebellopontine angle (CPA) component (figure 1). Consequently, a volumetric assessment of VS has been shown to be more accurate in determining their size and growth rate.^6^

The anatomical measurement of these vestibular tumours is complicated by their morphology. For instance, in the CPA their configuration is as a multilobulated mass of tumour surrounding the facial and cochlear nerves.^7^ Furthermore, there are typically two components to these tumours; one filling and bounded by the bony margins of the IAM itself and the second manifesting as a continuation into an adjoining CPA component (figure 1).

This morphology has often been discussed with regards to its nature and origin. Conventional thinking has for many years been that the tumours originate as solitary lesions at a point along the eighth cranial nerve called the junctional zone or glial–schwann cell junction, primarily thought to be on the superior division of the nerve.^7^ ^8^

Given the advances in high resolution vestibular nerve volumetric imaging, it is now feasible to assess more accurately the eighth cranial nerve in vivo and thereby validate these preceding hypotheses on vestibular schwannoma origin. As a national referral centre for children with NF2, we are able to perform standardised high-resolution imaging at an early time point in the disease process on a large cohort of children genetically proven to have NF2. Using this technique, we present the first report on the specific neural site of origin of these tumours and their relation to the vestibular nerve as a whole.

## Methods

### Study design and patient selection

This study was subject to local ethical review. Within the UK, the National Health Service has commissioned national groups to centralise the investigation, diagnosis, management and treatment of rare but significant disease processes. Neurofibromatosis Type 2 is such a commissioned service. The North of England and Scotland are served by the Manchester Unit, based in the Clinical Genetics Department at St Mary's Hospital, Manchester, UK. Paediatric patients are managed at the adjoining Royal Manchester Children's Hospital, one of the largest dedicated children's hospitals in Europe. All the imaging assessed in this study was acquired as part of this clinical management process.

### MRI acquisition and assessment

As part of the imaging assessment, all children referred to the NF2 service had dedicated MRI assessment of the brain and spine performed on a 1.5-tesla Avanto scanner (Siemens Healthcare, Erlangen Germany) using a dedicated head coil. Given the age range of our patient cohort, the methods for facilitating imaging vary, being determined by the clinical referral team in conjunction with radiology. Where sedation was necessary this followed routine protocol using chloral hydrate at 100 mg/kg child weight up to a maximum of 2 g dose. If necessary, patients were also referred for play therapy to facilitate successful imaging. All imaging was performed following the intravenous administration of a gadolinium-based contrast medium (Dotarem, Guerbet USA) at a dose of 0.2 ml/kg child weight which equates to 0.1 mmol/kg.

Specific to this study, three pulse sequences were employed: Coronal T1 weighted volume whole brain gradient echo sequence, TR 1160, TE 4.44, slice thickness 1 mm, field of view 230 matrix 192×256 with an acquisition time of 3 min 44 s. Axial and coronal whole brain spin echo T1 sequences with fat saturation, TR 556, TE 17, 3 mm slice thickness, field of view 220, matrix size 162×256 with an acquisition time of 6 min 55 s each.

Each MRI scan was reviewed by the same dedicated paediatric neuroradiologist. In cases where a finding was not definitive, confirmation was obtained from a second paediatric radiologist with an interest in neuroradiology. All imaging reviews were performed on a General Electric RA 1000 PACS workstation (GE Healthcare, USA).

### Image grading

**Figure 2:**
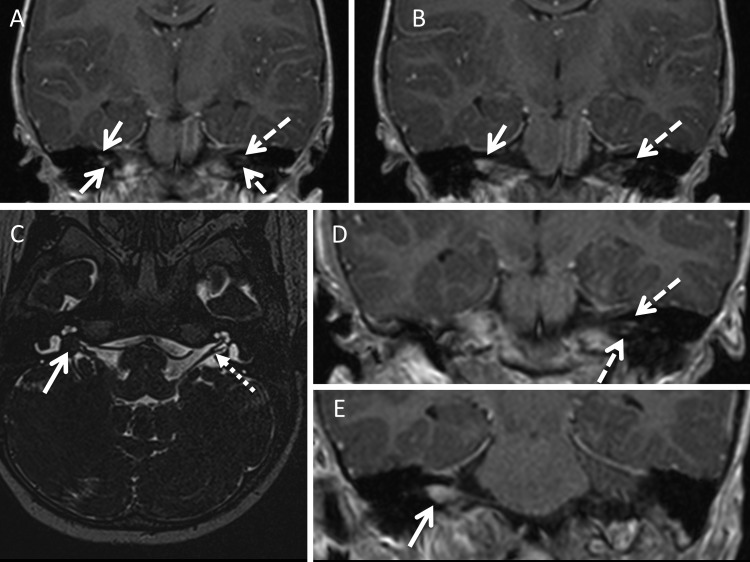
Coronal post contrast gradient echo sequence (A) in a 9-month-old child with failed hearing assessment demonstrates separate nodules of enhancement on the right superior vestibular nerve (SVN) and inferior vestibular nerve (IVN) (solid arrows) and the left (dashed arrows) SVN and IVN. The same sequence in the patient aged 2.5 years (B) shows the right side tumour foci to have grown, and while still being distinct on the nerve roots of origin, they are starting to collide/merge. The left sided appearances are static. By 4.5 years of age, the axial heavily weighted high-resolution T2 of the internal auditory meatus (IAM) (C) shows that there is now a discrete right sided IAM mass (solid arrow). The appearances of the left sided nerve roots in the IAM appear normal on this sequence (dotted arrow). The coronal gradient echo post contrast sequences (D and E) taken at the same age demonstrate once again a static appearance to the tumour foci on the left side (panel D, dashed arrows) while the right sided tumour foci have now coalesced into one tumour mass (panel E, solid arrow).

The T1 axial and coronal fat saturated images were used to give a general overview of any identifiable vestibular schwannoma. A measurement was made of the eighth cranial nerve along its course from the brainstem to the fundus of the IAM using the reformats to guide this measurement by tracing the course of the nerve in all three orthogonal planes. Measurement was made along the nerve to ascertain the length of the nerve from the point of its transition through the porus acousticus into the IAM and along to its termination at the fundus of the IAM. Points were then plotted along the nerves at the sites of tumour origin identified by foci of contrast enhancement (figure 2).

The position of the foci of tumour enhancement was then calculated as a percentage relative to the length of the nerve from the porus acousticus to the fundus of the IAM with a position at the porus being defined as 0% along the nerve length and a position at the fundus being 100% along the nerve.

### Statistical methods

The relationship between the number of tumours on the left and right sides of the head and between the IVN and SVN was assessed using Fisher's exact test on the tabulated data. The significance of the relative positions of tumours along the nerve was assessed using a Kolmogorov–Smirnov test for a uniform distribution in the relative position. p Values of less than 0.05 were considered significant and analyses were performed using R (The R Foundation for Statistical Computing, Vienna, Austria).

## Results

### Patient cohort characteristics

In total, 41 patients with genetically proven NF2 were assessed as part of the study: 59% boys and 41% girls. The mean age was 10.6 years (range: 1.0–19.0). Within the study sample, seven patients were found to have no identifiable vestibular nerve disease leaving 34 patients with measurable disease. The mean age of the disease-positive group was 11.4 years (range: 1.0–19.0).

### Laterality and discernibility of site of tumour origin

In patients where tumours were identified, only two patients presented with unilateral disease. In one patient there was a single point of tumour origin (with no disease seen on the opposite site) and in the second patient there was complete filling of the IAM with no disease seen on the opposite side. In all the other patients, bilateral disease was seen. The number of tumours and their laterality is shown in table 1.

**Table 1:** Tabulated data showing the number of tumours identified on both the superior and inferior vestibular nerves for each patient

Number of tumours	Left					Total
	None	1	2	3–5	Full	
Right						
None	7	0	0	4	0	11
1	1	0	1	1	0	3
2	0	0	3	1	0	4
3–5	0	2	0	5	1	8
Full	1	0	1	0	13	15
Total	9	2	5	11	14	41

‘Full’ implies the tumour mass could not be identified as a single tumour focus of origin and was filling the internal auditory meatus, with or without cerebellopontine angle encroachment. Fisher's exact test for independence of the numbers of left and right hand tumours is significant at p<0.001.

### Number of tumours and location

In 18 patients, the point of origin of the tumours could be localised to a specific site on the vestibulocochlear nerve in the CPA, or on the SVN or IVN in the IAM, and a total of 86 tumour foci were identified. In no cases did the tumour location comply with the conventional hypothesis of a single tumour focus on the superior division of the vestibular nerve alone.

**Figure 3:**
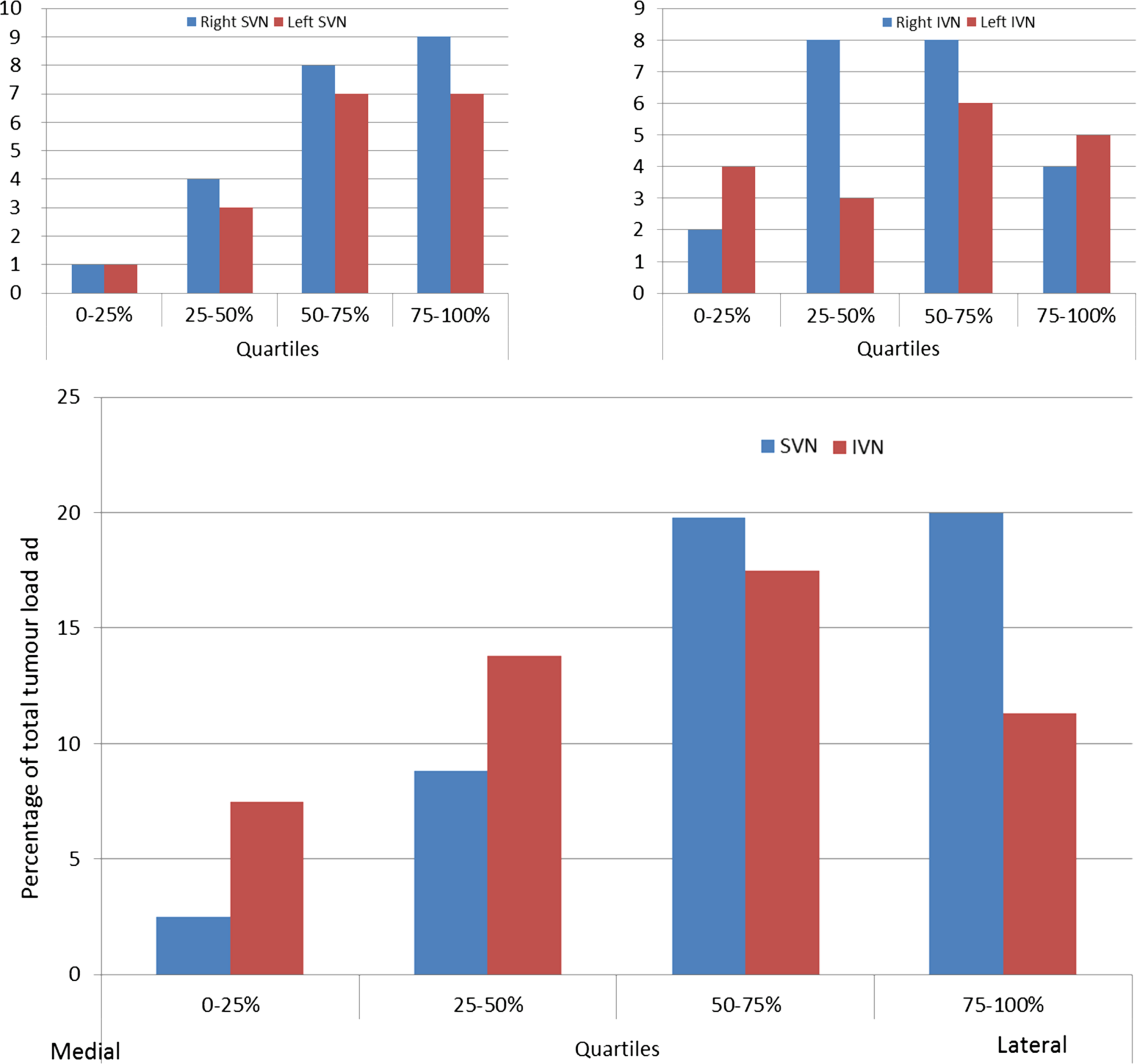
Graphical comparison of the sites of origin of tumours on the right and left superior vestibular nerve (SVN) (A), right and left inferior vestibular nerve (IVN) (B) and amalgamated results of all tumour sites as a percentage of total number of tumours (C). Tumours grouped and plotted as quartiles along the length of the SVN and IVN within the internal auditory meatus (IAM). A Kolmogorov–Smirnov test was significant for the SVN tumours (p<0.001) but not for the IVN tumours (p=0.11), both as two-sided tests, which indicates a statistical predominance for a tumour position towards the fundus of the IAM on the SVN alone.

In total, 23 tumours were seen on the right SVN in 17 patients with 26 tumours identified on the right IVN in 16 patients. In one case, the whole of the right IVN was seen to discretely enhance. In total, 18 tumours were found to originate from the left SVN in 12 patients with the left IVN being the site of tumour origin for 19 tumours in 8 patients. In addition there were two cases where both the left SVN and IVN were seen to enhance along their whole course. It was not possible to determine if these cases were true tumour foci or schwanomatosis. For this reason they were excluded from this analysis. In two cases (one on each side), a tumour was seen medial to the porus acousticus on the vestibulocochlear nerve within the CPA. Figure 3 shows the cumulative distribution of the positions of identified tumours along the SVN and IVN.

The relative position of the tumours on the SVN and IVN was calculated as (tumour position−porus position)/(total length−porus position). The two tumours before the porus were ignored. A Kolmogorov–Smirnov test was significant for the superior nerve tumours (p<0.001) but not for the inferior nerve tumours (p=0.11), both as two-sided tests which indicates a statistical predominance for a tumour position towards the fundus of the IAM on the SVN. The inferior nerve tumours were not displaced as far, although the positional difference between them and the superior nerve tumours (two sample Kolmogorov–Smirnov test) was not significant (p=0.55).

## Discussion

The radiological assessment of a child presenting with sensorineural hearing loss or clinical features of NF2 mandates early high-resolution imaging of the inner ear structures using MRI. This is usually in the form of a heavily T2 weighted axial acquisition (figure 2C). However, in the NF2 cohort a comprehensive assessment must include high-resolution post-contrast imaging as the evidence presented here clearly demonstrates foci of tumour enhancement along the components of the eighth nerves before discrete masses are demonstrated (figure 2). Some published works on sporadic eighth nerve schwannomas^8–13^ have demonstrated that there is no evidence for the commonly held belief that the tumour originates in the junctional zone, and in addition, resection specimens have shown an increased incidence of sporadic tumour in the inferior branch (84.8%) compared with the superior branch (8.9%) of the vestibular nerve based on 269 sporadic cases.^12^ This study demonstrates that in the NF2 cohort there is no predilection for tumour origin in either the superior or inferior branches of the vestibular nerves in NF2 with both branches being affected equally.

This study demonstrates that in addition to the bilaterality of vestibulocochlear disease in NF2, what can appear as a single tumour once established in fact comprises more than one focus of tumour originating at multiple sites on the SVN and IVN from the very earliest stages. In addition, contrary to the commonly held belief that the site of origin of VS is at the junctional zone, this study also shows that there is no specific site along the nerves within the IAM that has an increased incidence of tumour origin. There is a statistically significant finding that the sites of origin are more lateral on the SVN.

In sporadic VS, the lesion is generally a single tumour arising from the vestibular nerve at the porus acousticus. The adjacent facial and cochlear nerves are external to the tumour and displaced by it. We hypothesise that in VS these multiple sites of origin of VS go some way to explaining the difference in phenotypical description of a multilobulated tumour that appears like a ‘bunch of grapes’.^7^ ^8^ In NF2, as the tumours grow they eventually coalesce into one tumour mass. However, they retain their origin along the nerve such that the tumour bulk comprises an amalgamation of multiple tumour foci which envelope the facial and cochlear nerves. This explains the difference in the radiological phenotype seen as first postulated by Martuza and Ojemann.^8^ This is also likely to explain the lobulated pattern with the nerve fibres embedded in the tumour as shown histologically.^14^ ^15^ We also suggest that there is a correlation between the multiplicity of tumour origin and the aggressiveness of the overall NF2 disease process on an individual patient basis.

**Figure 4:**
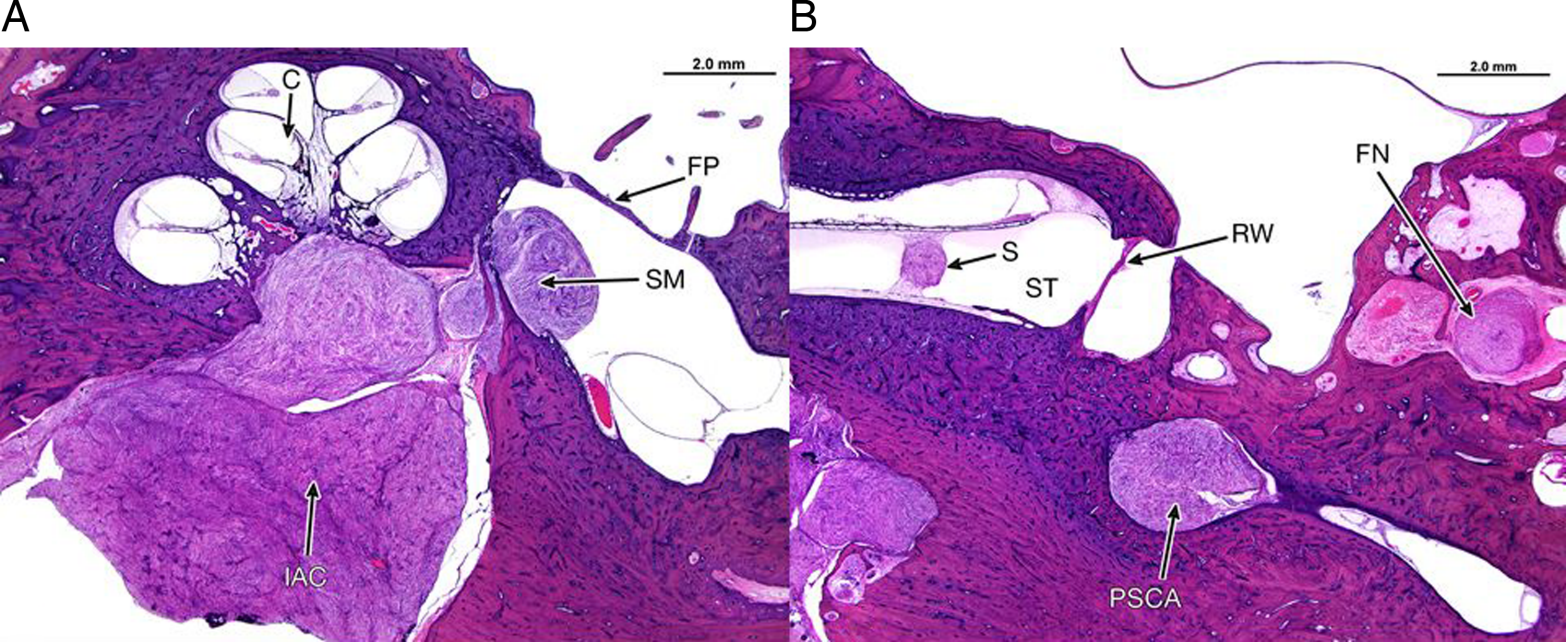
Temporal bone histology, H&E stain showing the multicentric origin of schwannomas in the internal auditory canal and labyrinth in a patient with neurofibromatosis Type 2. (A) An axial section of the right temporal bone through the cochlea (C). A large schwannoma is seen in the internal auditory canal (IAC) and a separate schwannoma arising from the saccular macula (SM) within the vestibule medial to the stapes footplate (FP). (B) An axial section through the right temporal bone at the level of the round window (RW). A small intracochlear schwannoma (S) is present within the fluid space of the scalatypmani (ST) and another schwannoma is present at the ampullated end of the posterior semicircular canal (PSCA). An additional, separate schwannoma arises within the descending segment of the facial nerve (FN).

We have also reported a separate tumour site involvement of the cochlear nerve and within the vestibule alongside multiple foci on the SVN and IVN, a finding that has significant clinical importance as it suggests that hearing and balance impairment could be related to separate involvement at these anatomical sites (figure 1) and not necessarily simply related to tumour size or growth along the tumour itself as currently thought. This is correlated by histopathological work in NF2 cohorts of patients referred for temporal bone resection. Histological studies of these resections confirm multicentricity of tumour foci on the eighth cranial nerve in 19/26 cases (73%) from 16 NF2 patients where multiple, separate, discrete tumour nodules were identified within the internal auditory canal (IAC) as well as within the labyrinth. Multiple tumour nodules were observed in the labyrinth involving the cochlea, vestibule or semicircular canals. The intralabyrinthal tumours were not a result of IAC tumour invasion which is therefore supportive of the imaging hypothesis of a multicentric tumour origin (figure 4).^14^

Furthermore, in a recent NF2 genetically engineered mouse model, generated through excision of the NF2 gene driven by Cre expression (restricted to periostin promoter element), the NF2 mice developed multiple schwannomas in peripheral and cranial nerves. Interestingly, as per this study, multiple, separate tumour nodules were present within the eighth cranial nerve, with multicentric origin of schwannomas identified in this mouse model.^16^

Outcomes from vestibular schwannoma microsurgery have long been shown to be inferior in patients with NF2 when compared with those from sporadic cases.^6^ ^17–19^ The higher tumour recurrence rates observed with both hearing preservation surgery and the trans-labyrinthine approach which more reliably removes all tumour suggest that a large proportion of these recurrences may be new primary tumours or tiny tumourlets that were not visible to the surgeon at the time of surgery.^17^ ^18^ The 59% recurrence rate in children who have undergone middle fossa cochlear nerve preserving surgery argues against this practice as a means of long-term hearing preservation even if both vestibular nerves are resected at the time of original surgery as does the presence of both facial and cochlear nerve schwannomas in temporal bone dissections.^14^ ^17^ ^20^

Several case series also suggest inferior rates of local control for NF2 as compared with sporadic VS after radiosurgery,^19^ ^21^ ^22^ although others have shown excellent results.^23^ Reasons for radioresistance of VS are poorly understood.^24^ If radiosurgery was undertaken for a very small NF2 vestibular schwannoma which had not yet reached the fundus of the IAM or the porus acousticus, then this article argues that careful consideration should be given to the treatment volume taking into account the possibility of multifocal lesions at the margins of the tumour, not identified on imaging, that may be receiving sub-therapeutic doses.

As discussed above, a radiological phenotype of bilateral IAM schwannomas relating to the eighth cranial nerves is pathognomonic of an underlying diagnosis of NF2 rather than the presence of a sporadic eighth nerve schwannoma.^8^ Additionally, we now suggest that the presence of an apparently isolated multifocal vestibular schwannoma should raise the strong possibility that the individual has a form of NF2.

In summary, this study refutes the origin of vestibular tumours in NF2 as simply being on the SVN, originating as a single tumour at the transition zone along that nerve. We also believe that it explains the later morphological appearance of these tumours in the older age group.

It is also hypothesised that there may be a relationship between the number of tumour foci and the aggressiveness of the disease process, as well as the likelihood of involvement of the facial and cochlear nerves. However, prospective longitudinal data obtained by a follow-up of the clinical outcomes in this cohort is necessary to illuminate this. Further work is also required to investigate whether underlying biological differences account for variation in both the number and location of vestibular schwannoma tumour foci seen within this NF2 patient cohort. It is largely conceivable that such differences would go some way to explaining why some of these children seem to respond to bevacizumab while others do not.

It remains apparent however that the development of targeted drug therapies such as bevacizumab are essential to improve quality of life and life expectancy in patients with NF2, particularly given the shortcomings of surgery and radiation treatments to deal with the multifocality of disease.
